# Influence of Framing: Recruitment to a Diabetes Disease Management Program From an Emergency Department Improves Enrollment and Outcomes

**DOI:** 10.7759/cureus.14116

**Published:** 2021-03-25

**Authors:** Rachel Moss, Emma K Craige, Brittany Levine, Mona Mittal, Seungjun Ahn, Brendan Appold, Mark Richman

**Affiliations:** 1 Emergency Medicine, Northwell Health Long Island Jewish Medical Center, New Hyde Park, USA; 2 Emergency Medicine, University of Virginia School of Medicine, Williamsburg, USA; 3 Emergency Medicine, Weill Cornell Medicine, New York, USA; 4 Preventive Medicine, Northwell Health Solutions, Great Neck, USA; 5 Biostatistics, University of Florida, Gainesville, USA; 6 Emergency Medicine, University of Michigan School of Medicine, Ann Arbor, USA

**Keywords:** diabetes, disease management program, dmp, emergency department, a1c, glycemic control

## Abstract

Introduction

Disease management programs (DMPs) provide education, self-management skills, care coordination, and frequent clinical assessment and medication adjustment. Our health system’s diabetes mellitus (DM) DMP recruited patients from an emergency department (ED) and outpatient settings (primary care physicians’ [PCP] and endocrinologists’ offices; cold calling patients with poorly-controlled diabetes). We investigated whether recruitment to a DMP from an ED is feasible and effective, hypothesizing such patients would have better enrollment rates, future A1c control, and ED utilization because their receptiveness to change was “framed” by their ED visit. “Framing” is the notion that the same problem presented using a different context impacts response to the information. Being told in an acute-care ED setting one has newly-diagnosed or poorly-controlled DM, or DM-related complications may influence desire/commitment to enroll in the DMP and make lifestyle/medication changes. That is, acute illness or acute setting may influence/”frame” willingness to enroll and improve glycemic control.

Methods

We captured all DMP recruitees’ demographic, medical, insurance, A1c, and recruitment venue characteristics and evaluated future enrollment rates, A1c, and ED utilization from any ED in our health system. We analyzed pre- vs. post-recruitment changes in A1c and ED visit rates, comparing patients recruited from the ED who enrolled, patients recruited from the ED who did not enroll, patients recruited from outpatient settings who enrolled, and patients recruited from outpatient settings who did not enroll. Continuous enrollment predictor and outcome variables were compared using the Mann-Whitney test; categorical outcome variables were compared using Fisher’s exact test.

Results

There were no statistically significant differences in characteristics (including mean baseline A1c [~11.4%]) among patients recruited from the ED, clinics, or cold calling. Twenty-five percent of all ED-recruited patients enrolled vs. 35% from outpatient settings. When a recruiter familiar with the DMP was in the ED, 41% of ED patients enrolled vs. 12% at other times (p=0.0001). Nearly 84% of ED visits were for direct DM-related causes (eg, diabetic ketoacidosis, hyperosmolar hyperglycemic state) or complications with a well-established link to diabetes (eg, acute coronary syndrome, stroke, wound infection); there was no statistically-significant difference in enrollment rates between patients whose ED visit was vs. was not for a DM-related complaint (53.8% vs. 60.0%, p=0.8018). No other variables, including whether the patient had newly diagnosed DM, were associated with enrollment. Enrollees with worse baseline glycemic control (A1c ≥11%) had a greater median A1c decrease (3.5% vs. 1.9%) vs. those with less-poor baseline glycemic control (A1c <11%) or those declining the program (p=0.05). Post-recruitment ED visits-per-patient-per-month decreased among patients recruited from the ED (-0.08), but not among those recruited from outpatient settings. (+0.08), p<0.0001).

Conclusion

ED recruitment to a diabetes DMP is feasible and effective. An ED-based diabetes DMP recruiter had enrollment rates substantially greater than a cold-calling DMP recruiter, comparable to enrollment rates from PCPs and endocrinologists, suggesting the importance of the recruitment framing/context. ED-recruited patients achieved substantial improvements in A1c and future ED visit rates.

## Introduction

Diabetes is associated with significant health care costs and is the third-leading cause of death in the United States (contributing to 11.8% of deaths) [1]. According to the American Diabetes Association, the total estimated cost of diagnosed diabetes was $245 billion in 2012, including $176 billion in direct medical costs and $69 billion in lost productivity [2]. An estimated 30.3 million Americans (9.4%) have diabetes. This includes 7.2 million people (23.8% of those with diabetes) who are undiagnosed and unaware of their disease [3]. Patients with diabetes are at greater risk of serious health complications, including heart attack, stroke, blindness, amputations, neuropathy, and renal failure. Those who receive early treatment can delay or prevent organ damage and death. However, significant barriers to effective treatment exist. As few as 36% of patients with diabetes are compliant with oral medications and 62% with insulin [4]. Misconceptions about diabetes are common, interfere with diabetes management, and lead to worse outcomes [5].

Effective care for patients with chronic diseases such as diabetes requires a multi-faceted, interdisciplinary approach as well as an educated, engaged patient making informed self-care, lifestyle, medication, and appointment compliance decisions. In the 1960s, Andersen developed the Behavioral Model of Health Services Use to understand healthcare utilization decisions [6]. The model shows healthcare decisions are made based on three domains: 1) predisposing domains (demographics, social issues), 2) enabling domains (transportation, social/community support), and 3) perceived need domains. Disease management programs (DMPs), which address one or more of the domains in Andersen’s model and are used widely in many healthcare organizations, are one approach to treating chronic conditions that have been shown to improve clinical and financial outcomes [7-9]. DMPs provide education on pathophysiology and methods of secondary prevention, self-management resources, counseling, case management services, care coordination, clinical assessment, shared decision making, and medication evaluation to decrease hyperglycemia and emergency department (ED) visits [10].

Approximately 10% of Emergency Department patients have undiagnosed diabetes according to studies performed in large EDs in Boston, New York, and Los Angeles; among those diagnosed with diabetes in the ED, half are non-compliant with their medications or follow-up appointments [11-13]. Given numerous ED patients with undiagnosed or poorly treated diabetes, many of whom present with acute diabetes-related illness, the ED would seem an ideal setting in which to screen for diabetes and enroll patients in a DMP. Despite this, few studies have examined whether ED diabetes screening, recruitment, and enrollment in comprehensive post-ED DMP care can impact long-term glycemic control and unscheduled care utilization (i.e., ED visits). “Framing” is the notion that the same problem presented using a different context or representation of information impacts the response to the information. Being told in an acute-care, unscheduled setting (i.e., the ED) one has newly-diagnosed diabetes mellitus (DM), or one’s long-standing DM is out of control or the cause of DM-related complications (cardiovascular disease, wound infections) may influence the patient’s desire and commitment to enroll in the DMP and make lifestyle and medication changes, as the patient is more-acutely aware of the significant medical, social, and financial (medical cost, and lost days of work) costs of poorly-controlled DM. Prior research has demonstrated the influence of message framing on health behavior changes. [14,15]

We investigated whether recruitment to a diabetes DMP from an ED is feasible and effective (both in terms of enrollment rates and of producing improvements in glycemic control and future ED visit rates). We hypothesized patients recruited from the ED would have better enrollment rates, future A1c control, and ED utilization, compared with those recruited from outpatient settings and cold calling, as the venue from which a patient was recruited may have influenced (“framed”) the decision to enroll. It is possible that the ED context “frames” patients’ understanding of the medical, social, and financial consequences of uncontrolled diabetes. Consequently, such patients may be more likely than patients recruited from other venues to enroll in the DMP or achieve improved glycemic control and fewer subsequent ED visits.

## Materials and methods

Northwell Health is a 22-hospital health system largely operating in Long Island and New York City. During the time covered by this study, Northwell’s diabetes DMP recruited adult patients with A1c ≥9 or ED visits in the past 12 months. Both patients with newly-diagnosed diabetes and those with established diabetes were eligible. Patients enrolling in the DMP received education, counseling, care coordination, clinical assessment, and medicine evaluation. Disease management services were provided by Certified Diabetes Care and Education Specialists (CDCESs) who were also Registered Dieticians (RDs), and a diabetes specialty nurse (RN). Each patient had recurrent visits (individual and group) with a specific CDCES/RD located in the patient’s primary care physician’s (PCP’s) practice Mondays through Fridays from 8 AM to 5 PM; RN visits were largely conducted by phone. The CDCES/RD kept in close communication with the PCP and endocrinologist, coordinating care and informing the physicians of the patient’s progress and needs (e.g., A1c above target, or markedly elevated fingerstick blood glucose levels). The program had no limit on time in the program or number of visits. However, as the patient’s glycemic control improved, the interval between DMP visits expanded. Depending on insurance status, DMP services were either no-pay or minimal co-payment for each CDCES/RD session. Patients were typically in the program 6-9 months; some were invited to remain longer if they achieved benefit and were determined to need longer care management on the basis of psychosocial concerns (e.g., behavioral health issues or vulnerable demographic characteristics, such as homelessness). 

**Figure 1:**
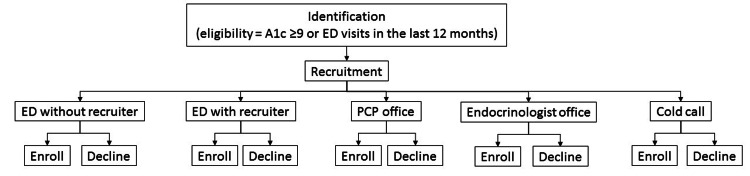
DMP recruitment and enrollment flow diagram ED: emergency department; PCP: primary care physician; DMP: disease management program

Patients were recruited to the DMP either at face-to-face primary care or endocrinology clinics, at ED visits, or via cold calling by a recruiter familiar with the DMP from a chase list of patients with elevated most-recent A1c results (Figure 1).

ED recruitment occurred at the Long Island Jewish Medical Center (LIJMC), a 500-bed teaching hospital in Queens serving a racially/ethnically and linguistically-diverse population of varied socio-economic status. This study examined data from the first 18 months of DMP operations (July 2016-December 2017). For 6 months (months 3-9) of operations, Northwell sponsored a DMP recruiter based in the ED. After month 9, the recruiter was deployed to other health system population health management activities and not replaced for the DMP.

Despite a reasonable or no cost for DMP services, not every patient recruited to the DMP enrolled. In accordance with guidelines from the Strengthening the Reporting of Observational Studies in Epidemiology (STROBE) project [16], we performed a retrospective investigation of which characteristics were associated with enrolling vs. declining the DMP. We then analyzed pre- vs. post-recruitment changes in A1c and ED visit rates, comparing patients recruited from the ED who enrolled, patients recruited from the ED who did not enroll, patients recruited from outpatient settings who enrolled, and patients enrollees recruited from outpatient settings who did not enroll. We hypothesized that, compared to decliners or outpatient recruitees, ED recruitees would have greater enrollment rates and reduced A1c and ED utilization, as acute illness may "frame" willingness to enroll and make lifestyle and medication compliance changes.

We retrospectively explored the association between potentially influential variables (demographic, medical, insurance, and recruitment site characteristics) and enrollment rates; and between recruitment site and outcomes (subsequent A1c and visits to any Northwell ED). For purposes of this investigation, all eligible patients (adult patients with A1c ≥9 or ED visits in the past 12 months) referred or recruited to the DMP were included. Patients who were referred but did not meet inclusion criteria (e.g., A1c <9 with no ED visits) were excluded. Analysis of pre- vs. post-recruitment A1c values was limited to patients in the program ≥3 months who had a post-recruitment A1c at least 3 months after recruitment. Because ED visits are, in general, uncommon for any given patient, analysis of post-recruitment ED visits was limited to patients who had been recruited at least 6 months prior to data collection, which allowed a reasonable time for a first or repeat ED visit, if any, to occur between recruitment and data collection. 

Research assistants unrelated to the DMP performed chart review using a standard template in a Health Insurance Portability and Accountability Act (HIPAA)-compliant database (REDCap, Vanderbilt University, 2018) to capture independent predictor variable data (demographic, insurance, and recruitment site characteristics) and dependent variable data (A1c, ED utilization). Continuous enrollment predictor and outcome variables were compared using the Mann-Whitney test; categorical outcome variables were compared using Fisher’s exact test. For all eligible recruitees, we examined enrollees vs. decliners from ED vs. outpatient (clinic and cold calling) recruitment site, looking at pre- vs. post-recruitment A1c and changes in ED visit rates. We sought to determine whether patients with higher initial A1c had greater declines in A1c than those with lower initial A1c, though we did not define, a priori, an A1c value that defined which group would benefit. Results were a priori considered statistically-significant at p<0.05.

This retrospective study was deemed exempt by the Institutional Review Board (IRB) (Human Subjects Research Determination (HSRD) 21-0005-LIJ).

## Results

Table 1 describes the ED-, clinic-, and cold calling-recruited populations. Half the patients were male; the average age was 53.8 years (78.1% were age 18-64 years); 72.6% were non-White; 21.9% stated their primary language was not English; two-thirds were on Medicaid; 14.3% had a new diagnosis of diabetes at the time of DMP recruitment; 71.3% had an additional major medical comorbidity (e.g., congestive heart failure); and 15.5% had a major psychiatric comorbidity (e.g., schizophrenia). There was no significant difference in the average A1c between patients from any recruitment venue who declined vs. enrolled in the program (p=0.19) (Table 1, bottom row).

**Table 1:** Demographic and baseline A1c characteristics of DMP recruited and enrolling patients ED: emergency department; DMP: diabetes management program

Characteristic	ED recruitee declined (N, %)	ED recruitee enrolled (N, %)	Clinic recruitee declined (N, %)	Clinic recruitee enrolled (N, %)	Cold call recruitee declined (N, %)	Cold call recruitee enrolled (N, %)	Total	%	P-value
Gender									
Male	51	13	37	23	6	3	133	50.2%	0.9267
Female	41	15	45	24	6	1	132	49.8%	
Age									
18 - 64	80	23	57	33	9	5	207	78.1%	<0.0001
≥65	11	5	23	18	1	0	58	21.9%	
Race									
White	19	6	13	9	7	3	57	27.4%	<0.0001
Black	42	12	38	17	1	0	110	41.5%	
Asian	12	5	15	7	2	0	41	19.7%	
Latino	18	6	15	16	1	1	57	58.2%	
Primary language									
English	72	19	66	37	8	5	207	78.1%	<0.0001
Spanish	16	5	9	11	1	0	42	15.8%	
Other	4	3	6	2	1	0	16	27.6%	
Insurance									
Medicaid	66	26	51	33	2	1	179	67.5%	<0.0001
Commercial/Medicare	30	6	23	14	9	4	86	32.5%	
Duration of diabetes									
New diagnosis of diabetes at recruitment	14	5	9	8	1	1	38	14.3%	<0.0001
Previously-established diabetes	78	22	71	42	9	5	227	85.7%	
Physical comorbidity									
Major physical comorbidity (eg, chronic kidney disease, congestive heart failure)	64	15	60	42	8	0	189	71.3%	<0.0001
No major physical comorbidity	29	12	21	8	3	3	76	28.7%	
Behavioral comorbidity									
Major behavioral comorbidity (eg, schizophrenia)	12	6	14	8	1	0	41	15.5%	<0.0001
No major behavioral comorbidity	80	21	67	42	9	5	224	84.5%	
Average most-recent A1c prior to DMP	11.7	11.6	11.4	11.4	11.4	11.6			

**Figure 2:**
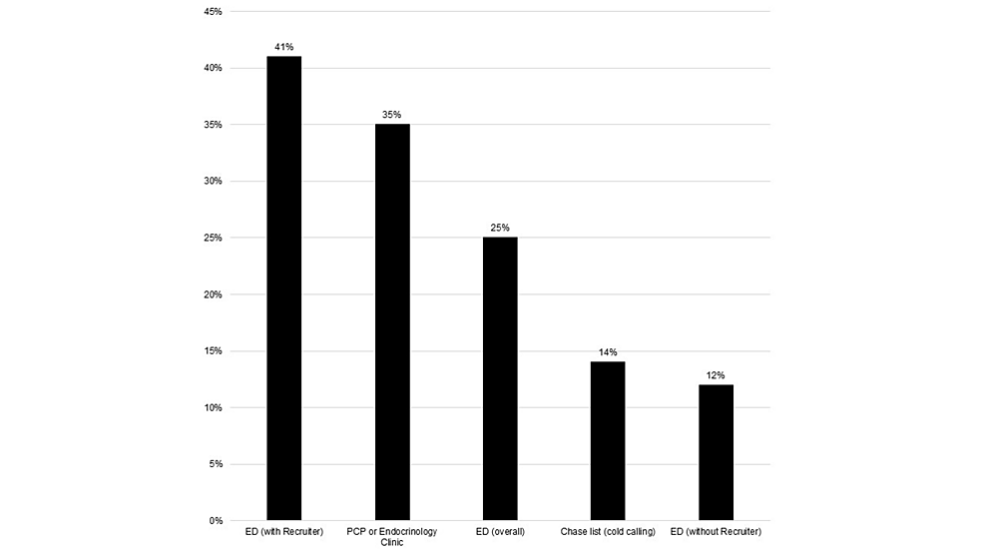
Percentage of recruited patients who enrolled in diabetes DMP, by recruitment site ED: emergency department; PCP: primary care physician

Twenty-five percent of 119 overall ED and 35% of 146 outpatient recruitees enrolled; 41% of ED patients recruited when a DMP recruiter was in the ED enrolled vs. 12% other times (p=0.0001). Only 14% of patients recruited by cold calling by a recruiter from a chase list enrolled (Figure 2, Table 2). Nearly 84% of ED visits were for direct DM-related causes (e.g., diabetic ketoacidosis, hyperosmolar hyperglycemic state) or complications with a clear link to diabetes (e.g., acute coronary syndrome, stroke, cellulitis, or wound infection); there was no statistically significant difference in enrollment rates between patients whose ED visit was vs. was not for a DM-related complaint (31.3% vs. 23.4%, p=0.1508).

**Table 2:** Comparison of enrollment rates between recruitment venues ED: emergency department

Comparison groups	P value (% difference, 95% confidence interval)
Outpatient (35%) vs. ED without recruiter (4%)	<0.0001 (31%, 19.2 - 39.5)
ED with recruiter (41%) vs. ED without recruiter (4%)	<0.0001 (37%, 22.7 - 49.2)
ED overall (25%) vs. ED without recruiter (4%)	0.0013 (21%, 9.4 - 30.0)
ED with recruiter (41%) vs. ED overall (25%)	0.023 (16%, 2.19 - 29.7)
ED with recruiter (41%) vs. Cold calling (14%)	0.0502 (27%, 0.05 - 42.5)
Outpatient (35%) vs. ED overall (25%)	0.0792 (10%, -1.2 - 20.6)
Outpatient (35%) vs. cold calling (14%)	0.1007 (21%, -4.7 - 33.4)
Cold calling (14%) vs. ED without recruiter (4%)	0.1635 (10%, -3.6 - 34.8)
ED overall (25%) vs. Cold calling (14%)	0.3474 (11%, -14.6 - 24.1)
ED with recruiter (41%) vs. Outpatient (35%)	0.3982 (6%, -7.5 - 19.9)

**Figure 3:**
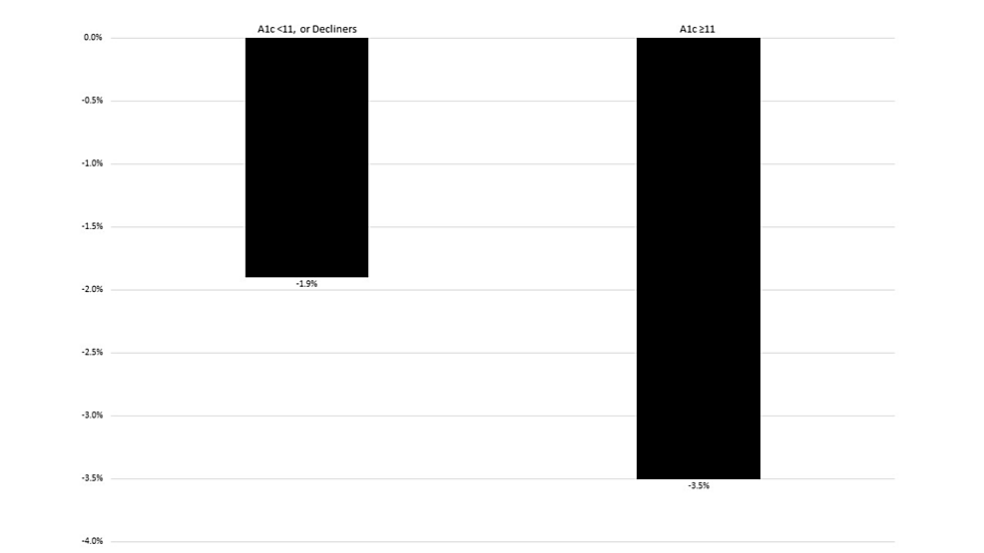
Post-ED recruitment change in A1c ED: emergency department

No other variables, including whether the patient had newly-diagnosed DM, were associated with enrollment. All recruitee sub-groups (ED vs. outpatient, enroll vs. decline) had similar mean baseline A1c (11.4%). A1c improvement was similar between overall ED (-1.9%) and outpatient (-2.1%) recruitees. However, ED and outpatient enrollees with baseline A1c ≥11% had median A1c decrease of 3.5% vs. 1.9% among patients with baseline A1c <11% or decliners (p = 0.05) (Figure 3)

**Figure 4:**
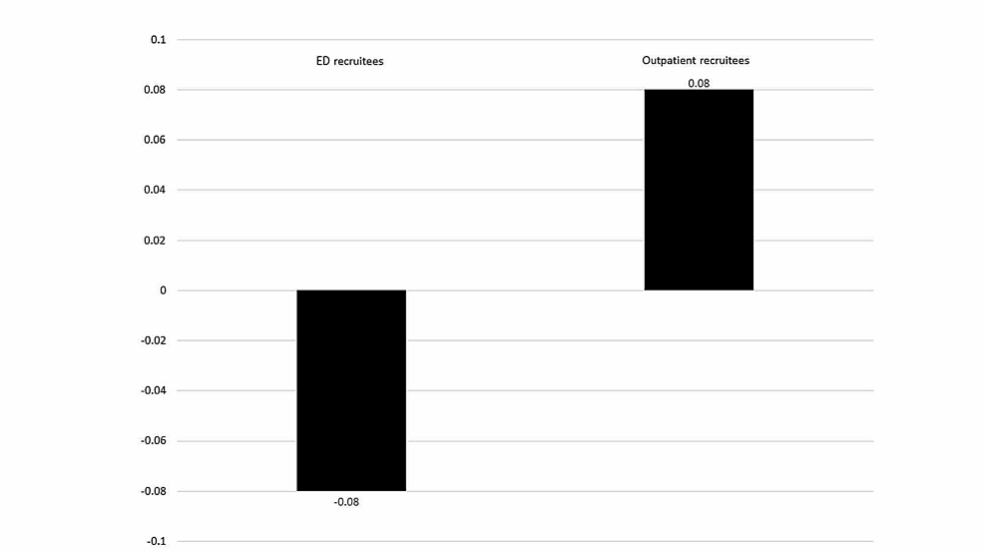
Post-recruitment ED visits per patient per month, by recruitment site ED: emergency department

Post-recruitment ED visits-per-patient-per-month decreased among ED enrollees (-0.08, n = 113) but not outpatient enrollees (+0.08, n = 131), p<0.0001) (Figure 3). There was a 5.9% decrease in total ED visits among ED-recruited patients vs. 1.3% increase among clinic-recruited patients (not statistically significant) (Figure 4).

## Discussion

The ED is an important and effective venue from which to recruit patients with diabetes to a DMP, from which they derive benefits in improved long-term glycemic control and decreased ED utilization. Recruitment using an ED-based recruiter was associated with a markedly higher enrollment rate compared to recruitment using a non-ED-based (i.e., office-based) cold calling recruiter (41% vs. 14% enrollment), and achieved comparable enrollment rates to PCPs or endocrinologists with whom the patient had a long-standing relationship (35%). In both circumstances (cold calling vs. ED recruitment), persons familiar with the DMP explained the program. The increased effectiveness of ED-based recruitment supports the idea that site-specific framing influenced enrollment decision. Although we did not ask patients their specific reason for enrolling, we hypothesize the acute nature of the patient’s diabetes-related ED medical problem (84% of patients), or its impact on their ability to engage in social activities or work, may have “framed” the problem in such a way that influenced their decision to enroll.

DMPs are associated with improved short- and long-term glycemic control (A1c reductions up to 0.75%), clinical outcomes, and annual ED utilization [7-9,17]. Patients enrolled in a DMP experienced, on average, $107.86 less costs per member per month compared to those not participating in this intervention; another study demonstrated a 30% decrease in costs. [18]

Despite these benefits, not every patient recruited to a DMP enrolls. Applying Andersen’s Behavioral Model of Health Services Use framework to DMP enrollment allows insights as to why this might be. According to Andersen, patients choose when and what kinds of healthcare services to use based on three domains: 1) predisposing factors (demographic characteristics, social issues), 2) enabling factors (transportation, social/community support), and 3) perceived need factors (e.g., severity) [9]. Successful recruitment addresses all three domains.

Our DMP recruitment program was designed with these domains in mind. Being from a minority ethnic background and without a 4-year college degree, the ED-based recruiter was relatable to our ED patients, who were of similar demographic characteristics (low-income [67.5% Medicaid], 22% non-English-speaking), potentially improving his recruitment success [19]. (Though we did not collect education level in our specific population, only 6% of the broader U.S. Medicaid population has a 4-year college degree [20].) The recruiter inquired about the patient’s social and financial circumstances, acknowledging the social determinants of health, demonstrating our program’s holistic concern, and building trust.

Our program addressed the many functional barriers within the “enabling” domain that may discourage DMP participation. Compared with “carve-out” DMPs (operated by an entity outside the patient’s health system), ours was “carve-in” (incorporated within the patient’s health system). “Carve-in” DMPs can be more appealing, making care more convenient and reducing the likelihood of healthcare fragmentation by utilizing patients’ PCPs and endocrinologists [21], and eliminating transportation and familiarity barriers. Our CDCES/RDs were located in patients’ PCP offices. Easy-to-use programs are favorable, since many patients to whom DMPs are targeted towards have low health literacy skills. Endorsement by someone the patient trusts (e.g., general practitioner or nurse) also encourages patient enrollment [22]. Not surprisingly, recruitment to our program was much more successful when performed by an ED-based recruiter dedicated to this task rather than “cold calling” or recruitment by ED providers not as familiar with diabetes education or our DMP. Our recruiter’s success rate was closer to the rate from the patients’ long-established, trusted outpatient sources of care (PCP or endocrinologist), highlighting the importance of dedicated, personable, “true believers” explaining and promoting the program.

In terms of perceived need domains, many patients explained they did not feel their disease was severe enough as a reason for declining enrollment in a DMP. Poor understanding of one’s condition correlates with low DMP enrollment and retention. Patients’ knowledge about risk factors, treatments, and the importance of self-care are key factors influencing willingness to participate [22]. The terminology used in recruitment is also important; patients told their conditions are “mild” are less likely to take active or urgent steps towards self-management [23]. Our enrollment criteria were: severe long-term hyperglycemia (A1c ≥9) or an ED visit due to a diabetes complication (e.g., hyperglycemia, diabetic foot infection) or lack of care coordination (e.g., poor primary care or endocrinology access, ran out of medications). The recruiter addressed perceived need factors by explaining the basics of diabetes, its complications, and why the patient’s condition was more advanced than mild. 

Our study has several limitations. This data was gathered from a single site study, operating only in the Northwell Health hospital health system; we may have missed ED visits to non-Northwell hospitals. It is also possible that changes in A1c and ED visits could have been due to regression to the mean. Furthermore, if an individual with an index ED visit does not have another ED visit within the next 6 months, it could simply be because, in general, people do not need emergency medical attention. Thus, the timeframe of this study may not have been long enough to capture a second rare event. Finally, some subgroups (e.g., cold-called patients) had small numbers, limiting comparisons.

## Conclusions

This was a retrospective study evaluating recruitment/enrollment into a diabetes DMP and clinical and utilization outcomes. Emergency department patients recruited to the DMP by a recruiter familiar with the program were more likely to enroll than those cold-called by a recruiter familiar with the program (enrolling at rates comparable to patients recruited by a PCP or endocrinologist) and achieve benefits in glycemic control (particularly among patients with the initially highest A1c) and future ED visit rates. There are two main takeaways to note from this study: 1) the importance of the venue/context in framing the importance of glycemic control, and 2) the effectiveness of a trusted, reliable program representative in turning recruitees into enrollees.
